# Bone shortening of clavicular fractures: comparison of measurement methods

**DOI:** 10.1186/s12891-017-1881-x

**Published:** 2017-12-19

**Authors:** A. H. Thorsmark, P. Muhareb Udby, I. Ban, L. H. Frich

**Affiliations:** 1 Department of Orthopedic Surgery, Zealand University Hospital, Køge, Denmark; 2 Department of Orthopedic Surgery and Traumatology, Hvidovre University Hospital: Orthopedic Research Unit, Hvidovre, Denmark; 3 Department of Orthopedic Surgery and Traumatology, Odense University Hospital: Orthopedic Research Unit, Odense, Denmark; 4 Department of Clinical Research, University of Southern Denmark, Odense, Denmark; 5 Department of Orthopedic Surgery and Traumatology, Holbæk Hospital, Holbæk, Denmark

**Keywords:** Clavicle, Fracture, Bone shortening, Measurement methods

## Abstract

**Background:**

The indication for operative treatment of clavicular fractures with bone shortening over 2 cm is much debated. Correct measurement of clavicular length is essential, and reliable measures of clavicular length are therefore highly requested by clinical decision-makers. The aim of this study was to investigate if three commonly scientifically used measurement methods were interchangeable to each other.

**Methods:**

A retrospective study using radiographs collected as part of a previous study on clavicular fractures. Two independent raters measured clavicle shortening on 60 patients using conventional radiographs on two separate sessions. The two measurement methods described by Hill et al. and Silva et al. were used on unilateral pictures. Side difference measurements according to Lazarides et al. were made on panoramic radiographs. The measurements were analyzed using intraclass correlation, Weir’s protocol for Standard error of measurement (SEM) and minimal detectable change (MDC), and Bland-Altman plots.

**Results:**

None of the methods were directly interchangeable. The side difference method by Lazarides et al. was the most reliable of the three methods, but had a high proportion of post-fracture bone lengthening that indicated methodological problems. The Hill et al. and Silva et al. methods had high minimal detectable change, making their use unreliable.

**Conclusion:**

As all three measurement methods had either reliability or methodological issues, we found it likely that differences in measurement methods have caused the differences in clavicular length observed in scientific studies.

## Background

Clavicular fractures are common and represent approximately 2.5–7% [1] of all fractures. Depending on the site and severity of the fracture, they are predominately treated non-operatively with good results. Various relative operative indications exist for mid-clavicular fractures, one such indication being post-fracture bone shortening above 20 mm0, [2–9] as conservative treatment has been linked to adverse outcome in terms of decreased strength and overhead motion of the arm. This is much debated, however, as a handful of studies have not been able to confirm these adverse results [10–13].

Essential for the correct treatment and classification of shortening is accurate measurement. However, there is no standardized method of measuring shortening, and different methods seem to have been used equivalently [3, 7]. The most commonly used measurement methods can be divided into two concepts: fragment overlap [3, 14] and side difference [7]. These two measurement approaches appear to be very different as they build on different concepts. The fragment overlap methods build on the principle of drawing a perpendicular line between the lateral and medial fragment. Shortening is then defined as distance from this line to the tip of the upper fragment. The side difference method uses the uninjured clavicle as reference. Shortening is then the difference in length from the injured clavicle length.

It is therefore very likely that measurement method used could influence the conclusion on shortening, and in the end be the cause for the debate about clavicular bone shortening. A previous study comparing different methods to measure shortening of *healed* clavicular fractures have shown that the estimated shortening varied significantly according to the method used [15]. Whether the estimated shortening on initial radiographs of *acute* displaced clavicular fractures is influenced by the measurement method is unknown. To investigate if the choice of measurement method could explain the different conclusions of peer-reviewed studies, we designed a validation study.

The aim of the current study was to compare three methods for measuring acute post-fracture mid-clavicular bone shortening with the objectives of describing measurement results by each method, estimating the inter- and intra-observer reliability and the inter-method agreement. The three methods were Silva et al. [14] and Hill et al. [3] (both based on the principles of fragment overlap) and Lazarides et al. [7](based on the principles of side difference).

### Ethical considerations

The study was a retrospective comparative study using radiographs that were collected as part of a not yet published study on clavicular fractures [ClinicalTrials.gov Identifier: NCT01483482]. The original study had been approved by the National Danish Data Registry (reference number: 2011–41-6031). Approval by the local ethical committee of the capital region was unnecessary.

## Method and power calculation

A power calculation was done using the formula, limits of agreement = $\sqrt{\frac{3}{n}}S$ = 16, where s is the standard deviation of the measurement difference between methods and n is the sample size. We wanted to estimate the limits of agreement within a margin of +/− 2.5 mm. We set the *s* as the normal anatomical standard deviation for clavicle length of approximately 10 mm [16] and found the number needed was 48 patients.

Of the 105 radiographs from the original study [ClinicalTrials.gov Identifier: NCT01483482], 25 were excluded due to non-accessible x-rays in the database and a further 20 were excluded because of incomplete panorama radiographs. The final study thus included 60 radiographs (60 patients) with acutely displaced clavicular fractures.

**Fig. 1:**
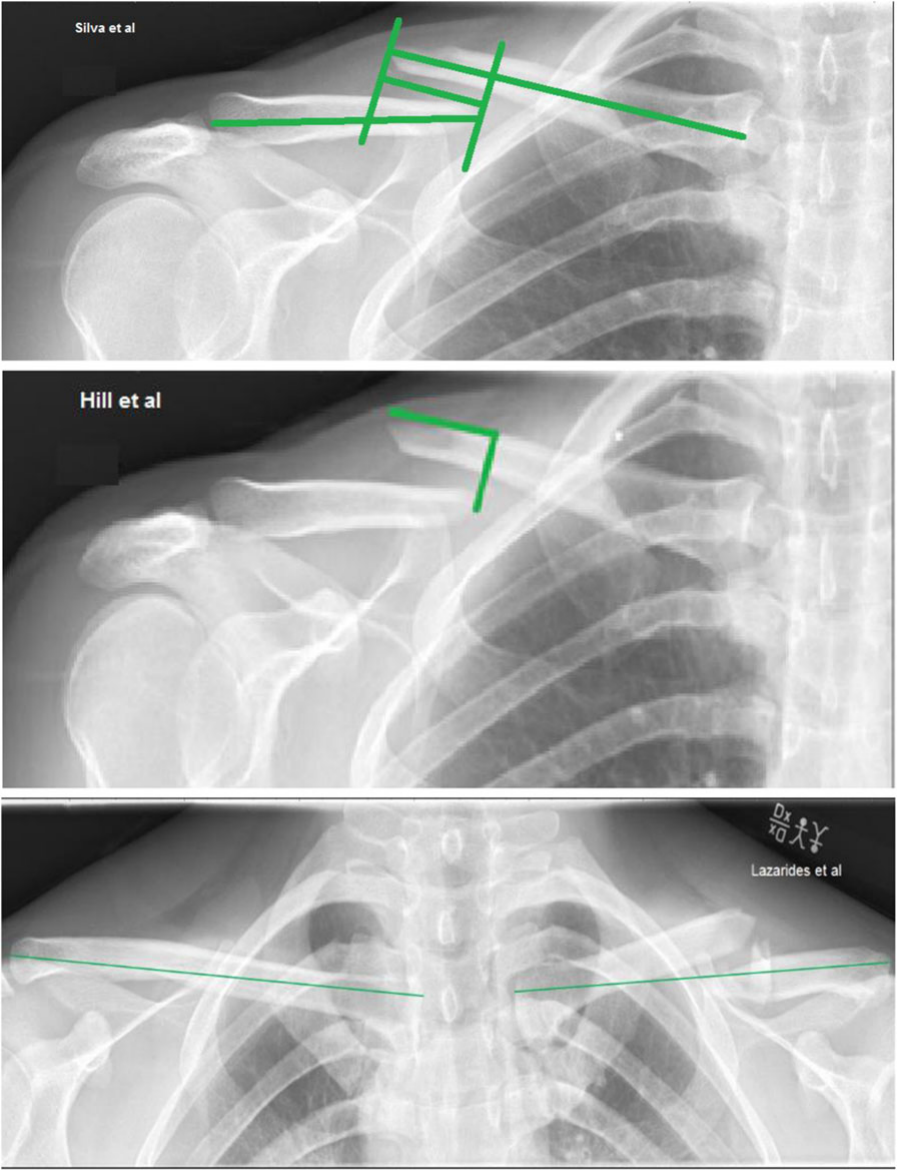
The three methods for measuring post-fracture clavicular length compared in this study. Silva [14]: a line is drawn through the middle of each fragment. From each middle line, a perpendicular line between each fragment is drawn. Bone shortening is defined as the distance between the perpendicular lines on single anterior-posterior view. Hill [3]: a line is drawn from the bottom fragment perpendicular to the top fragment. Bone shortening is defined from the line to the tip of the top fragment on single anterior-posterior view. Lazardis [7]: the length of each clavicle is measured. Bone shortening is defined as uninjured clavicle length minus injured clavicle length on a panorama view

Two raters measured the radiographs in five separate sessions at least two weeks apart. The raters were experienced junior consultants in orthopedics and trauma medicine who viewed the original articles for method instructions and agreed on how each measure was attained. During the sessions, the original studies were consulted for guidance if the raters were in doubt about the methodology, and the raters were blinded to each other’s results. The single anterior posterior radiographic view was used for the methods described by Silva et al. and Hill et al., while the panorama view including both clavicles was used for the method described by Lazarides et al. (Fig. 1). Length was measured using the available software (Carestream health inc. Verona street 150, Rochester NY 14608) which was noted in centimeters and converted to millimeters by the authors. For each radiograph, date and time were noted to ensure that the same radiograph was measured consecutively.

### Analysis

Statistical analysis was performed with use of STATA software (version 13.1; STATACORP, College Station, Texas). Simple descriptive statistics were used. Measurement distributions were assessed after dividing into three groups: lengthening (over 0 mm), neutral (between 0 mm and −19 mm) or clinical significant shortening (over 20 mm).

For comparison of reliability, we used the protocol described by Weir [17]. Inter-rater comparison used the second measurements made by each rater. The bone shortening intra-class correlation (ICC) was calculated for all three methods. A one-way random level for confidence was used for intra-observer reliability (one rater) while a two-way random level of confidence was used for inter-observer reliability (two raters). The obtained ICC values were used to calculate SEM, *Standard error of measurement* [SEM = SD × √ (1– ICC)] describing the given error for each measurement method. Afterwards MDC, *minimal detectable change*, was calculated with the use of SEM in the formula (MDC = 1.96 × √2 × SEM) to estimate the smallest given change each method would be able to detect.

Agreement between methods was visualized using Bland-Altman plots [18] estimating the convergent validity and limits of agreement comparing all three methods as reference.

Results

The final study group had a median age of 36.5 years (min. 18, max. 62) with 51 men and 9 women. Mean total length of the *pooled all measurements* clavicle for the *injured unilateral* clavicle was 160.8 mm (SD 14.9). For the *pooled all measurements panorama* radiographs, *uninjured* clavicle length was 168.2 mm (SD 12.8 mm) and *injured* 160.4 mm (SD 14.4 mm).

**Fig. 2:**
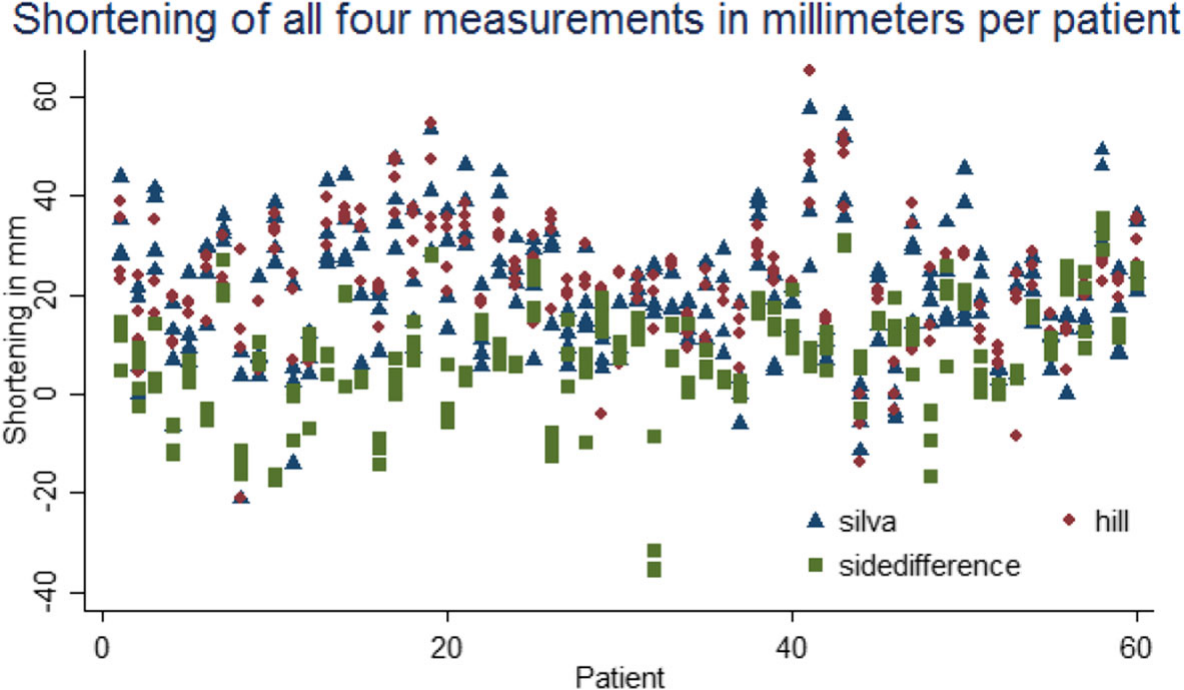
Plot of shortening as measured by the three respective measurement methods. Reference lines show 0 and −20 mm shortening. The side difference method by Lazarides et al. produced a large proportion of patients with lengthening of the bone after a fracture, while the Silva et al. and Hill et al. methods had a large proportion of patients with shortening over the clinical significant −20 mm

**Fig. 3:**
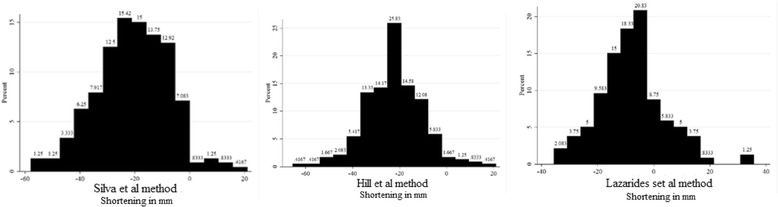
Histogram of shortening as measured by the three respective methods. The side difference method by Lazarides et al. found that 19% of all measurements had either lengthening or zero shortening. In comparison, the fragment overlap methods by Silva et al. and Hill et al. found that 4–5% of the measurements had either lengthening or zero shortening

The plot of the results (Fig. 2) from all 240 measurements (*n* = 60) from each method showed visually that the side difference method by Lazarides et al. was very different from the two fragment overlap methods, which were more similar to each other. The method by Lazarides et al. also found more patients with lengthening and fewer with shortening over 20 mm. Histograms (Fig. 3) showed that all three methods had a normal distribution pattern for the 240 measurements (*n* = 60). The Silva et al. method gave a mean shortening of −20 mm (SD: 13 mm), the Hill et al. method had a mean shortening of −22 mm (SD: 12 mm), and the Lazarides et al. method had a mean shortening of −8 mm (SD: 12).

When the measurements were divided into lengthening (over 0 mm), neutral (between 0 mm and −19 mm) or shortening (over 20 mm), the Silva et al. method showed that 5% of the measurements had lengthening, 43% were neutral and 52% had shortening. The Hill et al. method showed that 4% had lengthening, 31% were neutral and 65% had shortening. The Lazarides et al. method showed that 19% of the measurements had lengthening, 65% were neutral and 16% had shortening.

The three methods showed comparable ICC for both inter- and intra-observer reliability, with slightly better results for the Lazarides et al. method (Table 1) The mean bone shortening was comparable between Silva et al. and Hill et al., while Lazarides et al. had significantly shorter mean bone shortening. Standard error of measurement (SEM) was comparable for all three methods. The Silva et al. and Hill et al. methods had the largest minimal detectable change (MDC), while the method by Lazarides et al. had the smallest MDC of the three methods.

**Table 1:** The intra- and inter-rater reliability of post-fracture clavicular length measurement

	ICC	Mean (mm)	SD crude (mm)	SEM (mm)	MDC (mm)
Silva et al					
Intrarater 1	0.864	12.9	11.8	4.4	12
Intrarater 2	0.908	25.5	13.3	4	11
Interrater 1 and 2	0.724	20	13.8	7.4	20
Hill et al					
Intrarater 1	0.871	23.2	11.8	4.2	12
Intrarater 2	0.878	21.4	12.5	4.4	12
Interrater 1 and 2	0.768	21.7	11.9	5.7	16
Lazarides et al.					
Intrarater 1	0.942	7.8	11.8	2.8	7.9
Intrarater 2	0.945	7.7	12.0	2.8	7.8
Interrater 1 and 2	0.901	7.9	11.2	3.5	9.7

*ICC*: intra-class correlation. *Mean (mm)*: mean bone shortening in millimeters. *SD crude*: standard deviation of bone shortening in millimeters. *SEM (mm)*: standard error of measurement in millimeters. *MDC(mm)*: minimal detectable change in millimeters. For inter-rater analysis, the second measurement made by each rater was compared

**Fig. 4:**
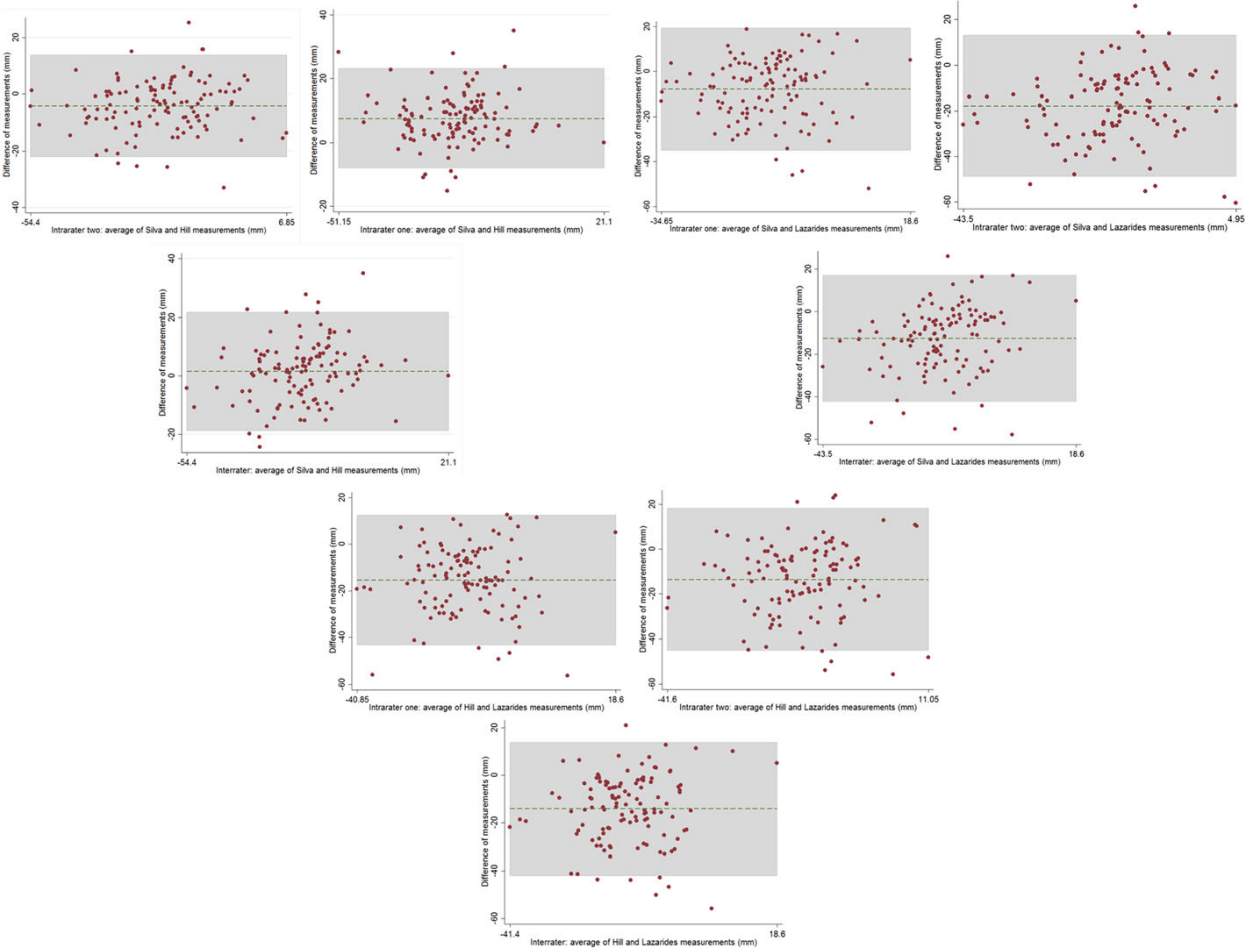
Bland-Altman plots of the three methods plotted against each other. Top: The Silva method versus the Hill method. The Bland Altman plot shows good agreement between the two methods but with reasonably wide limits of agreements. No graph is superior. Middle: the Silva method versus Lazarides method Bland Altman plots shows very poor agreement between the two methods as well as very wide limits of agreement. No graph is superior. Bottom: the Hill method versus Lazarides method. The Bland Altman plot shows very poor agreement between the two methods. Limits of agreement are very wide

Using Bland-Altman plots stating limit of agreement and mean disagreement, the fragment overlap methods (Silva et al. and Hill et al.) showed good consistency with each other but with very wide limits of agreement (Table 2, Fig. 4). Both Silva et al. and Hill et al. had very poor agreement with Lazarides et al., both in mean difference and limits of agreement (Table 2, Fig. 4).

**Table 2:** The inter-method agreement measurement of post-fracture clavicular length measurement

Intermethod agreement Bone shortening	Mean difference (mm)	Bland-Altman limits of agreement (LOA) – 95% CI
Silva vs Hill		
Intrarater 1	7.5 mm	- 8 mm to 23 mm
Intrarater 2	−4.2 mm	−22 mm to 14 mm
Interrater 1 and 2	1.5 mm	−19 mm to 22 mm
Silva vs Lazarides		
Intrarater 1	- 8 mm	−35 mm to 19 mm
Intrarater 2	−18 mm	−49 mm to 13 mm
Interrater 1 and 2	−13 mm	−42 mm to 17 mm
Hill vs Lazarides		
Intrarater 1	−15 mm	- 43 mm to 12 mm
Intrarater 2	−14 mm	−45 mm to 18 mm
Interrater 1 and 2	−14 mm	−42 mm to 13 mm

*Mean difference (mm)*; differences in mean when comparing two methods stated in millimeters. *Limits of agreement*; Limits stating the position of 95% of measurement. Results shown are a subsection out of 48 possible combinations. For interrater analysis the second measurements made by each rater was compared to each other

## Discussion

In this study comparing three previously described methods for defining bone shortening in acutely displaced mid-clavicular fractures, we found the side difference method by Lazarides et al. to differ substantially from the two fragment overlap methods, which showed good immediate comparability. Use of the side difference method resulted in more measurements showing lengthening and significantly fewer measurements with shortening over 20 mm. The side difference method also showed the best reliability based on standard error of measurement (SEM) and minimal detectable change (MDC). The two fragment overlap methods had a high MDC making their clinical use questionable, despite a respectable SEM. The fragment overlap methods by Silva et al. and Hill et al. had comparable mean differences, but with extremely wide limits of agreement i.e. 95% confidence intervals, while the measurements done by the side difference method by Lazarides et al. not in any way was correlated to the other methods.

Our results indicate that the measurement method chosen is critical to the measurement of post-fracture clavicular bone shortening, and studies involving clavicular bone shortening should be read with this in mind. Until now, different measurement methods have been described as equivalent, but our results show there is a clear distinction. The simple descriptive statistics, graphs and analyses showed that the side difference method described by Lazarides et al. gave results that were very different from the fragment overlap methods described by Silva et al. and Hill et al. This is also to be expected from the difference in measurement concepts.

A similar pattern was seen when analyzing the reliability of the methods. The fragment overlap methods by Hill et al. and Silva et al. were comparable regarding the standard error of measurement (SEM) and minimal detectable change (MDC), but these methods had very wide MDC*.* This suggests that their clinical use is unreliable for measurements purposes, as the minimal clinically important length is set to 20 mm – and it would not help to change to a more arbitrary limit e.g. 25 mm. In comparison, the Lazarides et al. side difference method showed a much better SEM and MDC*,* which should make it more clinically relevant. However, in this study we found methodological issues with the side difference method as it showed three times as many measurements with post-fracture *lengthening* of the bone than the fragment overlap methods. Lengthening after a fracture should theoretically be unlikely, as muscle pull would always tend to shorten the bone. These findings are probably a consequence of previously stated methodological issues with the side difference method, as it relies on the concept of bilateral length symmetry within individuals. A previous study have shown that this is not always the case, and clavicles follow a randomly distributed left and right length difference ranging between 0 and 15 mm [19]. Consequently, the bone lengthening observed in this study could be attributed to an underlying methodological error. Ultimately, even the use of the side difference method by Lazarides et al. is therefore problematic.

The limitations of this study are that measurements were done consecutively and only two raters were used. Any mistake or misunderstanding of the measurement methods could possibly be aggravated over a larger series of measurements. We tried to avoid this by regularly consulting the original description by Hill et al. or Silva et al. if in doubt. Our intra-class correlation was in fact slightly higher than results reported by Silva et al. [14] indicating that we were able to minimize this bias. A final limitation was that we had to exclude 45 of 105 available participants due to lack of images or errors on the radiographs. In this particular study, this was not of great importance as it was the rater agreement that was of interest and we managed to include 60 patients, which was well above our power calculation. We would not expect to see any significant change in the intra-class correlation if a higher number of participants were to be included. The strengths of this study are that is the first of its kind to compare intra-class correlation and to quantify SEM and MDC within and between clavicular bone shortening measurement methods.

## Conclusion

Our findings show that it is very likely that differences in measurement methods have caused the variation in results from studies on post-fracture clavicular bone shortening. Whether bone shortening results in adverse outcome is still subject to debate, but if used in a clinical setting it is important to have a reliable and accurate estimate. Based on our study, the side difference method by Lazarides et al. is the most accurate and reliable. However, as it relies on bilateral symmetry and identified a large proportion of patients as having bone lengthening rather than shortening, its use seems problematic. The two fragment overlap methods (described by Hill et al. and Silva et al.) appeared unreliable, and their use cannot be recommended. In conclusion, our findings raise a new question as to which method should be used, when taking both scientific and clinical grounds into consideration.

## Abbreviations

ICC: Intra Class Correlation
LOA: Limits of agreement
MDC: Minimal detectable change
SEM: Standard error of measurement
